# Evolutionary Origins and Functional Diversification of 2′-O-Methyltransferases: Insights from Phylogenetic and Structural Analysis

**DOI:** 10.3390/ijms26115260

**Published:** 2025-05-30

**Authors:** Sai-Nan Wang, Xiao-Xia Liu, Ling-Jie Lei, Qiang Wang, Zhu-Qing Shao, Yang Liu

**Affiliations:** School of Life Sciences, Nanjing University, Nanjing 210023, China; dg21300039@smail.nju.edu.cn (S.-N.W.); 502022300022@smail.nju.edu.cn (X.-X.L.); 502022300013@smail.nju.edu.cn (L.-J.L.); wangq@nju.edu.cn (Q.W.)

**Keywords:** RNA modification, methylation, 2′-O-methyltransferase, phylogenetic analysis, structural evolution, substrate adaptability

## Abstract

Ribose 2′-O-methylation (Nm), a key RNA modification, is catalyzed by diverse 2′-O-methyltransferases (2′-O-MTases), yet the evolutionary trajectories of these enzymes remain poorly studied. Here, with a comprehensive collection of functionally validated 2′-O-MTases, we classified them into 11 families based on the distinct methyltransferase (MTase) domains. Homology searches across 198 species identified 6746 proteins, revealing the widespread distribution of 2′-O-MTases across the Tree of Life. Eight MTase domains (e.g., FtsJ, SpoU-methylase) existed both in eukaryotes and prokaryotes, indicating their ancient origin in the Last Universal Common Ancestor (LUCA). In contrast, the AdoMet-MTase, TRM13, and Trm56 domains are lineage-specific. Copy number expansion of most 2′-O-MTase families occurred as life evolved from prokaryotes to eukaryotes, where they might engage in more complex regulation of cell differentiation and development. Domain composition, Ka/Ks ratio, and domain structural analyses showed that purifying selection conserved catalytic domains across most families, despite the frequent integration of auxiliary domains. Notably, the FtsJ family diverged into three deeply separated lineages via remodeling the catalytic pocket, with each lineage specializing in the methylation of mRNA caps, rRNA, or tRNA. These findings illuminate the evolutionary trajectory of 2′-O-MTases, highlighting their ancient multiple origins and functional diversification.

## 1. Introduction

RNA modifications, including N6-methyladenosine (m^6^A), 5-methylcytosine (m^5^C), 2′-O-methylation (Nm), N1-methyladenosine (m^1^A), N7-methylguanosine (m^7^G), N4-acetylcytosine (ac^4^C), and pseudouridine (Ψ), function as pivotal epigenetic regulators, orchestrating both co-transcriptional and post-transcriptional gene expression processes [1]. Among these, Nm is a highly abundant modification, occurs at the ribose sugar of nucleotides, and is found across multiple RNA types, including rRNA, tRNA, non-coding RNA, and mRNA [2].

In rRNA, Nm modifications stabilize the ribosome structure for translational accuracy [3], while in tRNA, they enhance tRNA thermal stability and decoding efficiency [4]. Small RNAs such as animal piRNAs and plant miRNAs acquire 3′-terminal Nm modifications to resist enzymatic degradation, ensuring their longevity [5]. In mRNA, 5′-cap Nm modifications not only stabilize the transcript but also help distinguish host transcripts from viral RNA, preventing immune sensor recognition [6]. Emerging evidence suggests that internal Nm in mRNA may influence translation efficiency and splicing, though the mechanisms behind this remain unclear [7,8].

Nm modifications are catalyzed by two distinct enzymatic systems: C/D-box small nucleolar ribonucleoprotein (snoRNP) complexes and guide RNA-independent 2′-O-MTases [2]. The Fibrillarin-containing C/D-box snoRNP complex catalyzes rRNA 2′-O-methylation in archaea and eukaryotes via a base-pairing process driven by guide RNA. To transfer methyl groups from S-adenosylmethionine (SAM) to the ribose 2′-OH at specified sites (e.g., 18S/28S rRNA in humans), Fibrillarin, the 2′-O-MTase, is positioned by the conserved C/D-box motifs and antisense element complementary to the target rRNA sequence found in the snoRNA component [3,9]. Guide RNA-independent methyltransferases, on the other hand, are able to directly identify the structural or sequence patterns of their substrates. For instance, TRM7 in humans binds the L-shaped tertiary structure of tRNA to create a heterodimer with Trm732 to methylate sites 32 and 34 in the anticodon loop, a process crucial to the precision of decoding [10]. Similarly to this, HEN1 catalyzes 3′-terminal 2′-O-methylation to prevent exonucleolytic degradation by recognizing the 2-nt 3′ overhang of plant miRNAs and animal piRNAs via a flexible loop that contains the FXPP motif (N-terminal to the MTase domain) [5]. The 5′-cap structure of mRNA in eukaryotes is subject to successive 2′-O-methylation: CMTR1 methylates the first transcribed nucleotide’s ribose 2′-OH, whereas CMTR2 alters the second nucleotide [11]. Since unmethylated cap0 structures are recognized by innate immune sensors (such RIG-I/MDA5) as pathogen-associated molecular patterns, this hierarchical methylation is essential for differentiating between viral and self-RNA [12,13]. Surprisingly, coronaviruses like SARS-CoV-2 imitate host CMTR1 methylation by taking advantage of a 2′-O-MTase complex (nsp16/nsp10) [14,15,16]. While nsp10 stabilizes the RNA-binding interface, the viral nsp16, which is structurally similar to CMTR1, methylates the first nucleotide’s 2′-OH utilizing SAM as a methyl donor and binds the cap0 structure via its K-D-K-E catalytic tetrad. Viral RNA can avoid host immune monitoring thanks to this mimicry, which is an example of an evolutionary arms race focused on 2′-O-MTase activity.

Fibrillarin is widespread across the Tree of Life, with mammalian Fibrillarin duplicating into two subgroups following the *Eutheria split*, suggesting potential functional diversification. Functional specialization resulting from this duplication has been demonstrated in *S. mediterranea* [17,18]. Phylogenetic analysis of the plant HEN1 protein family revealed several highly conserved motifs and intensive gene duplication in the evolution of the HEN1 family in flowering plants, which may be associated with whole-genome duplication events [19]. Similarly, evolutionary analyses suggest that at least one SPOUT member involved in 2′-O-methylation was present in the LUCA [20]. In addition to Fibrillarin, HEN1, and SPOUT, several other 2′-O-MTases have been found to participate in RNA 2′-O-methylation; these include TRM7 [21], FtsJ3 [22], TRM13 [23], and RSMI [24]; however, their evolutionary background and origins are still unclear. Understanding the development of Nm changes in various life domains requires a systematic evolutionary framework that includes a variety of 2′-O-MTases from the Tree of Life.

We report a phylogenetic analysis of 2′-O-MTases across 198 genomes from bacteria, archaea, and eukaryotes in this work, which involved a thorough collection and domain annotation of functionally proven 2′-O-MTases. We were able to determine their ancient origin and unique evolutionary patterns by combining domain architecture, selection pressure (Ka/Ks), and structural comparisons. Furthermore, we discovered that the FtsJ family was divided into three subclasses, each with unique substrate specificities and evolutionary-shaped structural characteristics. Our results present a thorough analysis of the history of 2′-O-MTases over a period of four billion years, offering fresh perspectives on their genesis and diversity.

## 2. Results

### 2.1. Literature-Based Collection of 2′-O-MTases Reveals 11 Distinct MTase Domains for Nm Catalyzation

**Figure 1:**
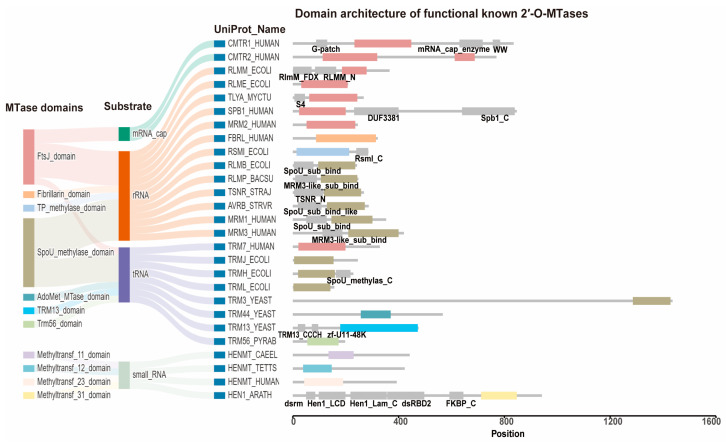
Domain annotation and classification of functionally known 2′-O-MTases. A total of 27 functionally characterized 2′-O-methyltransferases (2′-O-MTases) with distinct substrate specificities were retrieved from the literature. The UniProt Protein names of all proteins are provided, and full details including domain positions are listed in Supplementary Table S1. Based on domain composition, these enzymes were classified into 11 families, each sharing a conserved methyltransferase (MTase) domain. In the domain architecture diagram, colored domains correspond to the MTase domains shown in the first part of the Sankey diagram and are not duplicated. Gray segments represent other functional domains outside of the methylation-related MTase domain and are labeled directly in the figure. Domain annotations were obtained using HMMER3 with Pfam models, and domain names follow the InterPro convention.

To identify catalytic domains of 2′-O-MTases, we conducted a thorough survey of functionally known 2′-O-MTases reported in the literature. A total of 36 experimentally verified 2′-O-MTases were retrieved (Table S1), which represent 27 non-orthologous functional genes (Table S1). Hmmscan analysis revealed 11 distinct MTase domains from the protein sequences of these enzymes, including FtsJ (PF01728.24.hmm), Fibrillarin (PF01269.22.hmm), TP-methylase (PF00590.25.hmm), SpoU-methylase (PF00588.24.hmm), AdoMet-MTase (PF07757.18.hmm), TRM13 (PF05206.19.hmm), Trm56 (PF01994.21.hmm), Methyltransf-11 (PF08241.17.hmm), Methyltransf-12 (PF08242.17.hmm), Methyltransf-23 (PF13489.11.hmm), and Methyltransf-31 (PF13847.11.hmm), with domain names corresponding to the official Pfam annotations (Figure 1).

Among the 11 defined 2′-O-MTase families, most of them modify specific RNA substrates. For instance, TP-methylase modifies small rRNA [24], while TRM13 targets tRNA [23] (Figure 1). However, 2′-O-MTases with two domains exhibit broader specificity. For example, FtsJ family members catalyze Nm modifications on mRNA caps (CMTR1/2), rRNA (RLMM/RLME/TLYA/SPB1/MRM2), and tRNA (TRM7). Similarly, SpoU-methylase modifies both rRNA and tRNA (Figure 1).

Interestingly, an extra RNA-binding domain called SpoU-sub-bind (like) is typically present in the SpoU-methylase family members that exclusively catalyze rRNA Nm alterations (Figure 1). On the other hand, SpoU-methylase family members that modify tRNA usually have a simpler domain design that just includes the SpoU-methylase domain (Figure 1). The DHU loops 42 and 43 (TRMH/TRM3) and the anticodon loops 44 and 45 (TRMJ/TRML) are examples of fixed loops where the target tRNA modification sites for SpoU-methylase proteins are frequently found [25,26,27,28]. However, the FtsJ family did not exhibit any other substrate-related domains. Surprisingly, the various proteins in this family can identify their particular substrates with just the FtsJ domain—no additional RNA-binding domains are required. According to this, the FtsJ domain could have developed an innate method for identifying a broad range of RNA targets, allowing for significant substrate diversity and adaptation across various RNA types.

### 2.2. Phylogenetic Distribution of the 11 MTase Domain-Containing Proteins Suggests Ancient and Life Kingdom-Specific Origins of 2′-O-MTases

**Figure 2:**
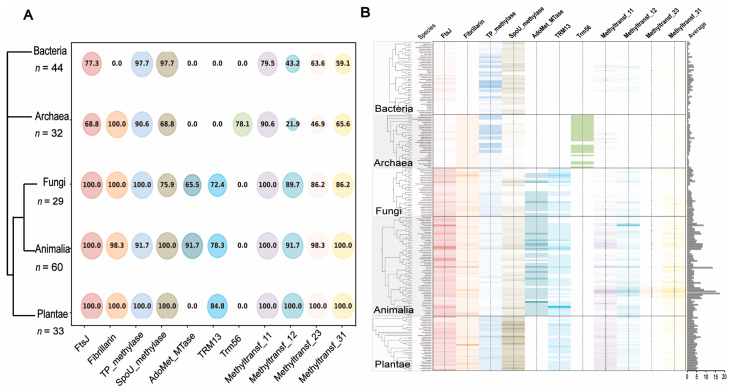
Phylogenetic distribution of the 11 2′-O-MTase families across the Tree of Life. (**A**). The proportion of the investigated species containing each of the 2′-O-MTase family genes across the bacteria, archaea, fungi, animals, and plants. Color-coded bubbles represent 11 families, with size indicating the proportion of species containing each family (largest circle = 100%). (**B**). Abundance of different 2′-O-MTases in each species. Colors intensity indicating relative quantity. Gray bars show the average number of 2′-O-MTases per species.

For 11 MTase domains identified from 2′-O-MTases, we analyzed their distribution across 198 species from the Tree of Life, including 19 bacterial groups (44 species), 14 archaeal groups (32 species), 10 fungal groups (29 species), 23 animal groups (60 species), and 9 plant groups (33 species). The results show that proteins containing the FtsJ, SpoU-methylase, TP-methylase, and Methyltransf-11, -12, -23, and -31 domains are widely distributed across eukaryotes and prokaryotes, suggesting that these 2′-O-MTase families originate from the LUCA. Notably, Fibrillarin is conserved across eukaryotes and archaea. The TRM13 family is absent from bacteria and archaea, indicating its origin in the common ancestor of eukaryotes. The AdoMet-MTase family is present in fungi and animals, and the Trm56 family is specifically found in archaea (Figure 2A).

Generally, the total 2′-O-MTase gene copy number increased during the prokaryote-to-eukaryote transition, reflecting the growing complexity of RNA modifications (Supplementary Figure S1). Among the eight families present in both prokaryotes and eukaryotes, FtsJ, SpoU-methylase, and Methyltransf-11, -12, -23, and -31 families show an overall increase, the Fibrillarin family is relative stable, and TP-methylase is enriched in prokaryotes and plants (Figure 2B, Table S3).

### 2.3. Diverse Domain Architectures of 2′-O-MTases

**Figure 3:**
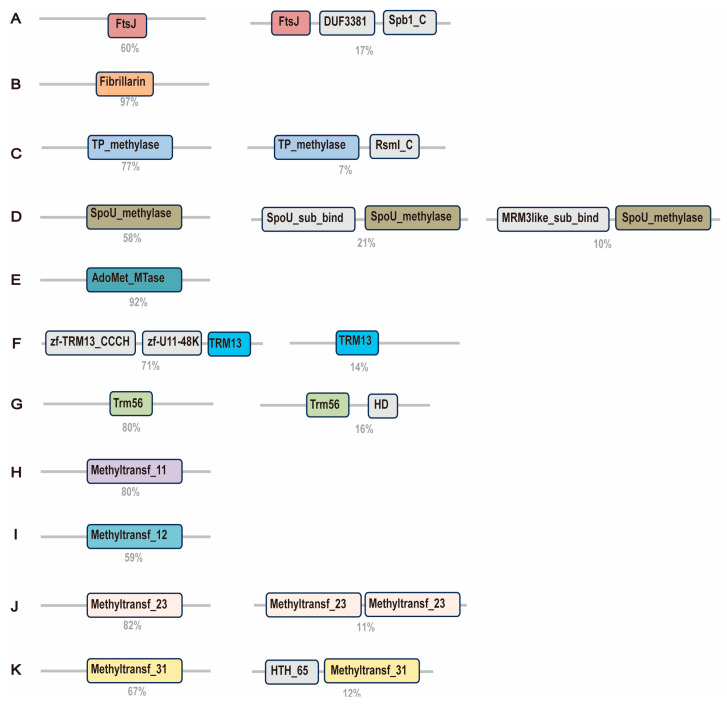
This figure illustrates the domain architectures of each 2′-O-MTase family (**A**–**K**). Each sub-figure shows the domain architectures for a specific 2′-O-MTase family. Colored domains represent the 11 MTase domains that we studied, while gray domains represent other domains. Only the combinations that account for 10% or more of the total proteins in each family are shown.

To explore functional diversification among 2′-O-MTases, we analyzed domain architectures across 11 families. AdoMet-MTase, Fibrillarin, and Trm56 exhibit the minimal domain complexity, with most proteins maintaining a single-domain (Figure 3A–K, Table S4). In contrast, the FtsJ family frequently combines the core MTase domain with the Spb1-C and DUF3381 domains (Figure 3A), which are essential for ribosome assembly [29]. SpoU-methylase often fuses the SpoU-sub-bind domain, likely enhancing rRNA binding specificity (Figure 3D). The four Methyltransf families exhibit complex domain architectures, potentially linked to diverse regulatory mechanisms (Figure 3H–K, Table S4).

### 2.4. Evolutionary Features of Sequence and Structure of 2′-O-MTases

**Figure 4:**
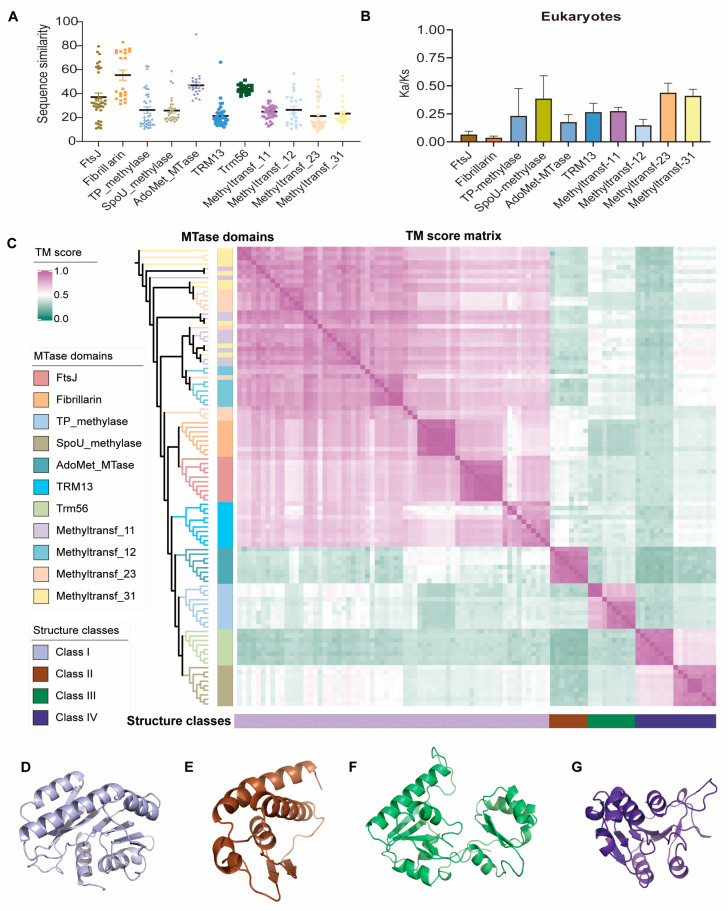
Sequence similarity, selective pressure, and structural similarity of 2′-O-MTases. (**A**). Sequence similarity of 11 2′-O-MTases. Colored scatter points represent pairwise similarity values across families. (**B**). Selective pressure of each 2′-O-MTase family in eukaryotes. (**C**). Structural similarity and clustering of 11 2′-O-MTase families across 10 species. Matrix plot shows pairwise TM-scores, with color intensity indicating structural similarity. (**D**). Representative structure of Class 1 (NP_497843.1_Caenorhabditis-elegans_FtsJ). (**E**). Representative structure of Class 2 (NP_741176.2_Caenorhabditis-elegans_AdoMet_MTase). (**F**). Representative structure of Class 3 (WP_031175390.1_Streptomyces-albus_TP_methylase). (**G**). Representative structure of Class 4 (NP_495375.3_Caenorhabditis-elegans_SpoU_methylase).

An integrated analysis comprising sequence similarity analysis (Figure 4A, Table S5), selective pressure analysis (Figure 4B, Table S6), and structural similarity analysis (Figure 4C, Table S7) was carried out to investigate the evolutionary trends of 2′-O-MTases. In eukaryotes, all 10 families showed Ka/Ks ratios consistently below 1 (Figure 4B), despite differing levels of sequence divergence (Figure 4A), suggesting a general purifying selection. Among the 11 2′-O-MTases, Fibrillarin exhibited exceptional sequence conservation (an average of 57.95% sequence similarity cross different life kingdoms and 75.92% among eukaryotes), ultralow Ka/Ks ratios (0.0259–0.0546), and high structural conservation (average TM score = 0.85), indicating strong purifying selection on the catalytic core, consistent with its essential role, while gene duplication likely led to regulatory diversification (see Discussion). The TRM13 and Trm56 families, on the other hand, showed relaxed purifying selection (Ka/Ks: 0.21–0.34) and moderate sequence plasticity (11.1–41.9% similarity).

The hierarchical clustering resolved 2′-O-MTases into four conserved structural classes, each with a TM score average of 0.74. These are Class I: Methyltransf-11, -12, -23, and -31, FtsJ, Fibrillarin, and TRM13; Class II: AdoMet-MTase; Class III: TP-methylase; and Class IV: SpoU-methylase and Trm56 (Figure 4C). Class I enzymes typically feature a seven-stranded β-sheet surrounded by α-helices, forming an open αβα sandwich structure. Class II enzymes usually have a two-stranded β-sheet along with three α-helices. Class III enzymes possess a bilobal structure. Class IV enzymes typically possess a six-stranded parallel β-sheet and seven α-helices, with the C terminus forming a unique “knot” structure (Figure 4D,E). Notably, within Class 1, we observed striking spatial structural similarity across distinct families. For example, subclades of Methyltransf-11, -12, -23, and -31 exhibited intermixed distributions with an average TM-score of 0.82, while FtsJ and Fibrillarin shared high structural similarity (average: 0.84). Given that structural evolution is slower than sequence divergence [30], these findings suggest that these families may share a common evolutionary origin, despite their sequence-level divergence.

**Figure 5:**
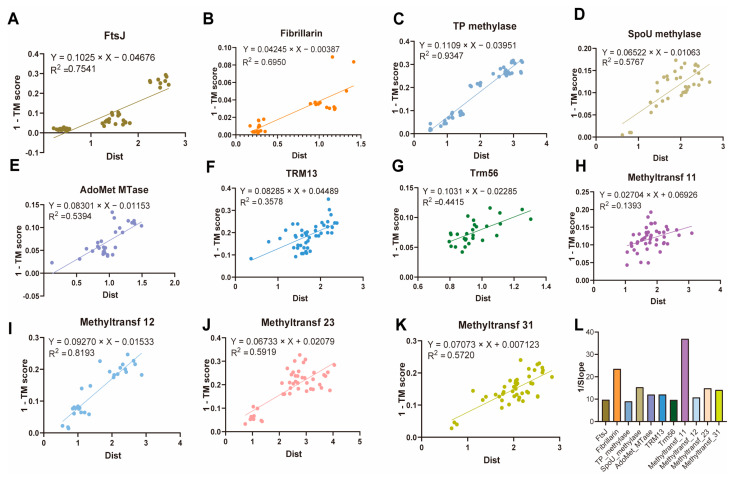
Structural sensitivity of 2′-O-MTases. (**A**–**K**). Structural distance versus evolutionary divergence in 11 2′-O-MTase families. Scatter plots showing the relationship between structural distance (1-TM score) and evolutionary divergence (amino acid substitutions per site) for (**A**) FtsJ, (**B**) Fibrillarin, (**C**) TP-methylase, (**D**) SpoU-methylase, (**E**) AdoMet-MTase, (**F**) TRM13, (**G**) Trm56, (**H**) Methyltransf-11, (**I**) Methyltransf-12, (**J**) Methyltransf-23, and (**K**) Methyltransf-31. Correlation equations and R² values are shown for each family. (**L**). Bar plot of 1/slope values for each family.

Furthermore, we computed the correlation between structural divergence (1-TM score) and sequence evolutionary distance across 2′-O-MTase families in order to evaluate the link between sequence evolution and structural conservation. Structural changes trail sequence divergence, as evidenced by the strong positive correlation (slope < 1; Figure 5A–K). The inverse slope (1/slope), which is a measure of structural tolerance, identified conservation tactics unique to each family: Fibrillarin maintained high structural similarity (structure similarity score > 0.8) across kingdoms, even with moderate sequence divergence (∼58% sequence similarity), demonstrating strong structural robustness (1/slope = 23.55; Figure 5L). The Trm56 (1/slope = 9.70) and TRM13 (1/slope = 12.07) families, on the other hand, showed constrained plasticity, allowing for substantial sequence variation (11.1–41.9% similarity) while maintaining structural integrity.

### 2.5. Ancient Divergence and Substrate-Adaptive Evolution of the FtsJ Protein Family

**Figure 6:**
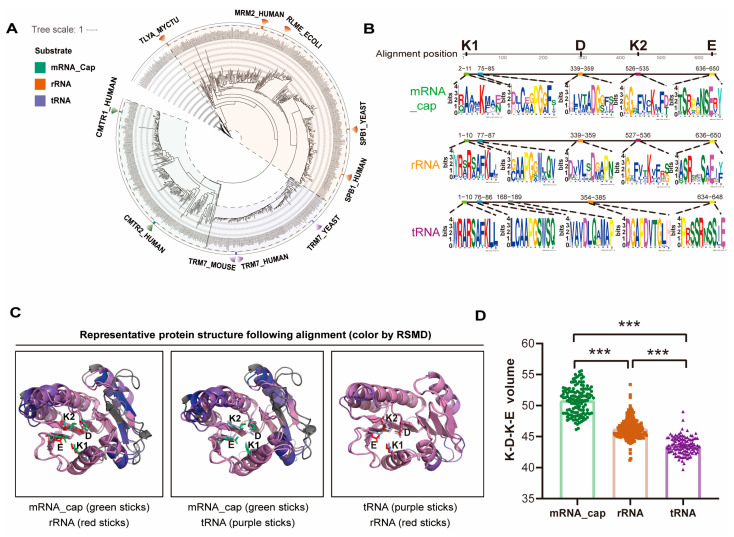
Subclass-specific features in FtsJ family. (**A**). Phylogenetic analysis of FtsJ subclasses. Maximum likelihood tree shows the evolutionary relationships among mRNA-cap (green, *n* = 191), rRNA (red, *n* = 316), and tRNA (purple, *n* = 139) subclasses. (**B**). Motif analysis of FtsJ subclasses. Sequence logos depict conserved motifs, with the catalytic tetrad (KDKE) highlighted. (**C**). PyMOL-generated alignments of representative proteins: mRNA-cap (Q8N1G2-CMTR1-HUMAN), rRNA (Q8IY81-SPB1-HUMAN), and tRNA (Q9UET6-TRM7-HUMAN). Structures are colored according to per-residue RMSD: pink indicates regions with low RMSD (high structural similarity), blue denotes regions with high RMSD (greater structural divergence), and gray highlights structural elements present in only one of the aligned proteins. The KDKE catalytic tetrad residues are displayed in stick representation. (**D**). Catalytic pocket volume across FtsJ subclasses. Bar plot compares KDKE pocket volumes for mRNA-cap, rRNA, and tRNA subclasses. Error bars represent the SEM for each subclass. *** indicates *p* < 0.001.

As previously mentioned in Figure 1, despite having identical individual domain designs, the FtsJ family proteins show extraordinary substrate variety, with several members performing modifications on rRNA, tRNA, and mRNA-cap. Phylogenetic analysis of 679 FtsJ-domain-containing proteins from 198 species revealed three deeply separated lineages (Figure 6A). It is interesting to note that FtsJ proteins with varying substrate specificities for rRNA, tRNA, and mRNA-cap were found in three separate lineages, indicating that lineage divergence may have contributed to the development of substrate specificity in FtsJ proteins. This pattern suggests that FtsJ proteins may have been specialized for rRNA, tRNA, and mRNA cap modification as a result of each lineage’s adaptation to certain substrate types.

Motif analysis showed that all FtsJ subclasses retain the essential KDKE catalytic core but exhibit subclass-specific features (Figure 6B). As previously reported, K1 stabilizes the RNA phosphate group, D binds SAM, K2 facilitates nucleophilic attack, and E coordinates K1 and K2 [31,32,33,34]. Despite KDKE conservation, each subclass exhibits unique sequence characteristics. For instance, the mRNA-cap subclass is characterized by a distinctive residue composition, with K1 frequently forming the RAAMK motif. On the other hand, the RSAFK motif is commonly formed by both the rRNA and tRNA subclasses. In contrast to the other subclasses, the tRNA subclass notably exhibits greater sequence conservation, indicating a potentially more stringent structural requirement for its target changes. These varied evolutionary paths highlight the FtsJ domain’s structural flexibility, which permits functional adaptability to a variety of RNA substrates while maintaining basic catalytic activity.

**Figure 7:**
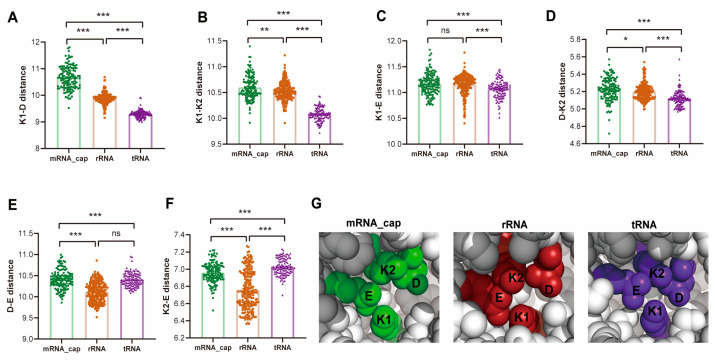
Subclass-specific structural variations in the KDKE catalytic tetrad in the FtsJ family. A-E. Distance analysis of KDKE catalytic tetrad residues. (**A**) K1-D, (**B**) K1-K2, (**C**) K1-E, (**D**) D-K2, (**E**) D-E, and (**F**) K2-E distances across FtsJ subclasses: mRNA-cap (green), rRNA (red), and tRNA (purple). Distances were calculated based on Cα atoms. (**G**) Structural alignment of FtsJ subtypes. PyMOL-generated alignments of representative proteins: mRNA-cap (Q8N1G2-CMTR1-HUMAN, green), rRNA (Q8IY81-SPB1-HUMAN, red), and tRNA (Q9UET6-TRM7-HUMAN, purple), shown as spheres. The KDKE tetrad is highlighted in each structure. Error bars represent the SEM for each subclass. * indicates *p* < 0.05, ** indicates *p* < 0.01, *** indicates *p* < 0.001, and ns indicates not significant.

Structural prediction and alignment revealed high intra-subclass consistency but notable inter-subclass divergence, particularly in the N-terminal regions of mRNA-cap modifiers outside the conserved KDKE catalytic core (Figure 6C). These variations likely enhance substrate recognition, driving functional diversification. Quantitative analysis of the KDKE catalytic tetrad showed subclass-specific features: mRNA-cap modifiers exhibited the largest catalytic cage volume (Figure 6D), accompanied by an expanded K1-D spacing (Figure 7A), whereas the rRNA/tRNA subclasses retained compact configurations. These structural differences may reflect evolutionary adaptations to substrate size and complexity—the enlarged cage of mRNA-cap modifiers accommodates the bulky 5′ cap, while the narrower pocket in tRNA subclasses matches the anticodon loop (Figure 7A–G).

## 3. Discussion

2′-O-methylation, catalyzed by 2′-O-MTases, is essential for RNA stability and cellular regulation. This study classifies 2′-O-MTases into 11 families, revealing their widespread distribution and distinct evolutionary trajectories. The results reveal that 2′-O-MTases expanded considerably from prokaryotes to eukaryotes, where they may be involved in regulating differentiation and development [35,36].

Among the 11 2′-O-MTase families, the Fibrillarin family exhibits remarkable evolutionary conservation both in sequence and structure, reflecting its essential role in rRNA 2′-O-methylation. Despite moderate sequence divergence (~58% similarity across kingdoms), Fibrillarin maintains high structural similarity (TM-score > 0.8), suggesting strong structural robustness. This robustness likely reflects strong purifying selection that preserves the conserved structural fold critical for its catalytic function, while still allowing for peripheral sequence variation. Although gene duplication is typically associated with functional divergence, Fibrillarin homologs—such as FBLL1 in mammals or fbl-1/fbl-2 in planarians—exhibit divergent tissue-specific expression patterns while retaining highly conserved MTase domains [18,36]. This suggests that functional diversification occurred primarily through regulatory divergence and changes in non-catalytic regions (e.g., the GAR domain), rather than alterations in the catalytic core. Therefore, strong purifying selection on the MTase domain coexists with subfunctionalization, enabling specialization without compromising essential enzymatic activity.

SpoU-methylase and FtsJ modify multiple substrates through distinct strategies. Specifically, SpoU-methylase incorporates additional domains (such as a SpoU-sub-bind-like module for rRNA targeting), whereas FtsJ becomes diversified through structural adaptations. For the FtsJ family proteins, phylogenetic analysis categorizes them into three subclasses specified for mRNA-cap, rRNA, and tRNA, respectively, each characterized by unique catalytic pocket sizes. Among them, mRNA-cap modifiers have the largest pocket for cap accommodation, rRNA modifiers maintain intermediate sizes for complex folding, and tRNA modifiers feature compact pockets for anticodon loop modification. These structural variations highlight the role of flexibility in the functional diversification of the FtsJ family, driven by evolutionary pressures to fine-tune substrate specificity.

Spatial structural analyses reveal that the catalytic domains of some 2′-O-MTases exhibit remarkable structural similarity during evolution, suggesting a potential origin from a common ancestral fold [37]. Despite sequence divergence obscuring direct phylogenetic relationships, the slower-evolving structural features retain detectable evolutionary signatures, as evidenced by the four conserved structural classes observed, which hint at a diversified origin for early 2′-O-MTase modification systems. However, the possibility of functional convergence cannot be excluded—similar structures may have arisen independently due to convergent evolution under shared catalytic geometric constraints. Future studies could resolve the homology versus convergence problem by dissecting conserved motifs (e.g., substrate-binding pockets), thereby elucidating how RNA modification tools from the LUCA era have evolved to underpin modern biological complexity.

Collectively, this study uncovers the ancient origin of diverse 2′-O-MTases, establishing a framework for understanding how RNA modification systems have become diversified to regulate biological complexity across different life domains.

## 4. Materials and Methods

### 4.1. Identification of 2′-O-MTase-Conserved, Methyltransferase-Associated Domains

To systematically identify RNA 2′-O-MTase-conserved, methyltransferase-associated domains, we conducted a comprehensive literature search using the keywords ‘RNA 2′-O-methylation’, ‘Nm methylation’, and ‘RNA 2′-O-methyltransferase’, focusing on enzymes validated by in vivo, in vitro, or knockdown experiments (Table S1). We then performed hmmscan analysis (HMMER v3.1b2) [38] on these known 2′-O-MTases with stringent parameters (-E 0.01 --domE 0.01 --incE 0.01) to identify conserved MTase domains.

### 4.2. Identification of 2′-O-MTases Across the Tree of Life

To analyze the distribution of 2′-O-MTases across different life domains, we selected 198 genomes from 44 bacterial [39,40], 32 archaeal [39,41], 29 fungal [42,43], 60 animal [44,45], and 33 plant species [46,47], representing well-sequenced organisms across life kingdoms (Table S2). Using previously identified 2′-O-MTase-related MTase domains, we performed a genome-wide hmmsearch (-E 0.01, --domE 0.01, --incE 0.01) to identify 2′-O-MTase family members. Sequences were manually curated to ensure completeness, with truncated sequences excluded.

Data retrieval and analysis utilized NCBI (https://www.ncbi.nlm.nih.gov/, last accessed on 20 May 2024), JGI (https://genome.jgi.doe.gov/portal/, last accessed on 28 May 2024), GTDB (https://gtdb.ecogenomic.org/, last accessed on 8 June 2024), and Phytozome (https://phytozome-next.jgi.doe.gov/, last accessed on 12 June 2024). Annotation files (GFF/BED) were used to filter isoforms, retaining the longest gene version for analysis.

### 4.3. Domain Architecture Pattern Analysis

Domain annotation for 2′-O-MTase homologous proteins was performed using hmmscan (-E 0.01, --domE 0.01, --incE 0.01). Domain architectures were manually verified for completeness and functional relevance. We analyzed frequently occurring (appearing >2 times) and functionally validated architectures and examined the co-occurrence and frequency of MTase domains with other domains.

### 4.4. Multiple-Sequence Alignment and Phylogenetic Analysis

Based on the hmmscan results, the MTase domain sequences from each of the 11 protein families were extracted. Pairwise alignments were then performed using Clustal Omega v1.2.4 (default parameters) [48] to calculate their sequence similarity.

To resolve phylogenetic relationships within the FtsJ family, we performed multiple-sequence alignment using Clustal Omega v1.2.4. The phylogenetic tree was constructed using IQ-TREE2 [49] with the optimal substitution model selected automatically by IQ-TREE2 using the ‘-m TEST’ option (1000 bootstrap support). Experimentally validated proteins were mapped onto the phylogeny to analyze their functional distribution across FtsJ subclasses.

### 4.5. Ka/Ks Analysis

The protein sequences and coding sequences (CDSs) of each 2′-O-MTase domain were aligned and converted into codon-based alignments using ParaAT v2.0 [50], respectively. KaKs-Calculator 3.0 [51] was used to estimate nonsynonymous (Ka) and synonymous (Ks) substitution rates, as well as the Ka/Ks ratio for each alignment.

### 4.6. Structural Similarity Analysis and Clustering

To assess selective pressures on 2′-O-MTases from a structural perspective, we performed sequence and structural distance comparisons of representative protein domains. Protein structures were predicted using ColabFold (3 recycles, Amber relaxation) [52], and domains with a mean pLDDT score greater than 70 were extracted. TM scores were calculated using US-align [53]. Protein sequence distances were determined with MEGA12 (JTT model, 1500 bootstraps) [54], and structural distances were calculated as 1-TM-score. Linear regression was used to analyze the relationship between structural similarity and evolutionary distance [30], with adjusted R² indicating the strength of the correlation and 1/slope representing evolutionary structural tolerance (Table S7). Pairwise structural similarities (1-TM-score) were used to generate a distance matrix. Hierarchical clustering was performed with PHYLIP-Neighbor to construct NJ trees [55].

### 4.7. Polyphony Analysis

Polyphony is a differential geometry approach suitable for analyzing flexible protein structures. To perform the spatial structural classification of 2′-O-MTases, we prepared the structural data according to the literature [56]. We first used the polyphony_create_fasta_file.py code from the Polyphony suite to generate sequences and corresponding residue-level structural data. Then, we performed sequence alignment using MAFFT [57] with the parameters --localpair and --maxiterate 1000 to ensure accurate alignment of the protein sequences. Finally, we used the integrated polyphony_Ete2_tree.py code, which incorporates multiple modules from the Polyphony project, including Structural_Alignment, Properties, Structure_Matrix, and Tree, to handle tasks such as structural alignment, property calculation, structural difference matrix generation, and tree construction. These steps enabled us to conduct a comprehensive structural analysis and classification of the 2′-O-MTases family.

### 4.8. Motif Identification

To identify conserved motifs in the FtsJ family, we used the MEME Suite under the ZOOPS model, allowing for flexible motif detection [58]. The analysis identified up to five motifs (4–10 residues wide), sorted by their aligned positions in subclass multiple-sequence alignments.

### 4.9. Tetrahedron KDKE Volume Calculation and Pairwise Distance Analysis

To quantify the KDKE catalytic motif volume, we performed multiple-sequence alignment of the MTase domain of FtsJ family proteins, identifying KDKE positions through alignment with experimentally validated active sites. The α-carbon (Cα) atoms of K1, D, K2, and E were extracted for structural analysis. The tetrahedral volume of the KDKE catalytic cage was calculated using the formula $V=\frac{1}{6}\left|\vec{v_{1}}\cdot \left(\vec{v_{2}}\times \vec{v_{3}}\right)\right|$. Here, $\vec{v_{1}}$ is the vector from point $p_{K1}$ to point $p_{D}$, $\vec{v_{2}}$ is the vector from point $p_{K1}$ to point $p_{K2}$, and $\vec{v_{3}}$ is the vector from point $p_{K1}$ to point $p_{E}$. Additionally, pairwise distances between the Cα atoms of the KDKE residues were calculated using the Euclidean distance formula: $d=\sqrt{\left\{{\left(x_{2}-x_{1}\right)}^{2}+{\left(y_{2}-y_{1}\right)}^{2}+{\left(z_{2}-z_{1}\right)}^{2}\right\}}.$ Here, $\left(x_{1},y_{1},z_{1}\right)$ and $\left(x_{2},y_{2},z_{2}\right)$ are the coordinates of the two sites.

## 5. Conclusions

Based on unique MTase domains, we categorized 2′-O-MTases into 11 families based on a collection of functionally reported 2′-O-MTases. Overall, 6746 homolog proteins were identified via a domain-based search against 198 species in all living kingdoms, indicating that 2′-O-MTases have been widely distributed throughout the evolution of life. Of the 11 families of 2′-O-MTases, 8 were widely spread in both eukaryotes and prokaryotes, while the remaining ones were life domain-specific. Purifying selection has allowed for conservative evolution of the catalytic domain at both the sequence and structural levels, despite the fact that the integration of additional domains occurred frequently throughout the evolution of most 2′-O-MTase families, according to domain composition, Ka/Ks ratio, and domain structural analyses. In conclusion, our research sheds light on the evolutionary history of 2′-O-MTases, emphasizing their numerous ancient origins and functional diversity.

## Data Availability

Data are contained within the article and Supplementary Materials.

## Supplementary Materials

The following supporting information can be downloaded at https://www.mdpi.com/article/10.3390/ijms26115260/s1.

## Abbreviations

The following abbreviations are used in this manuscript:

Nm	2′-O-methylation
2′-O-MTase	2′-O-methyltransferase
MTase	Methyltransferase
LUCA	Last Universal Common Ancestor
Ka	Nonsynonymous substitution rate
Ks	Synonymous substitution rate
m^6^A	N6-methyladenosine
m^5^C	5-methylcytosine
m^1^A	N1-methyladenosine
m^7^G	N7-methylguanosine
ac^4^C	N4-acetylcytosine
Ψ	pseudouridine
SAM	S-adenosylmethionine
piRNA	Piwi-interacting RNA
miRNA	MicroRNA
snoRNP	Small nucleolar ribonucleoprotein
