# Supplementary material for: Evolutionary Origins and Functional Diversification of 2′-O-Methyltransferases: Insights from Phylogenetic and Structural Analysis
Source: Int J Mol Sci. 2025 May 30;26(11):5260. doi: 10.3390/ijms26115260 (PMC12155479; doi:10.3390/ijms26115260)
Supplement: Supplementary file 1 [file ijms-26-05260-s001.zip › Supplementary_Figures.pdf]

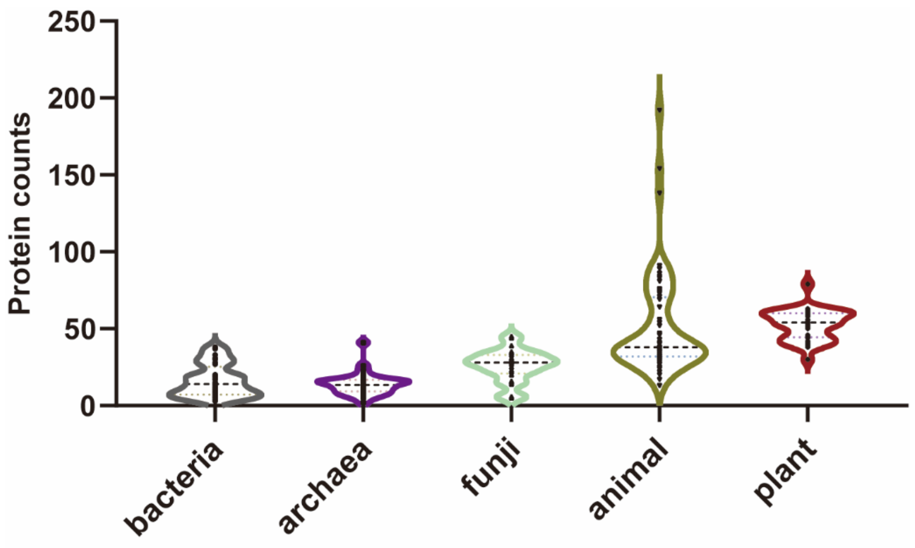

Supplementary Figure S1. Protein counts of all 2′-O-MTases homologs in different life kingdoms.

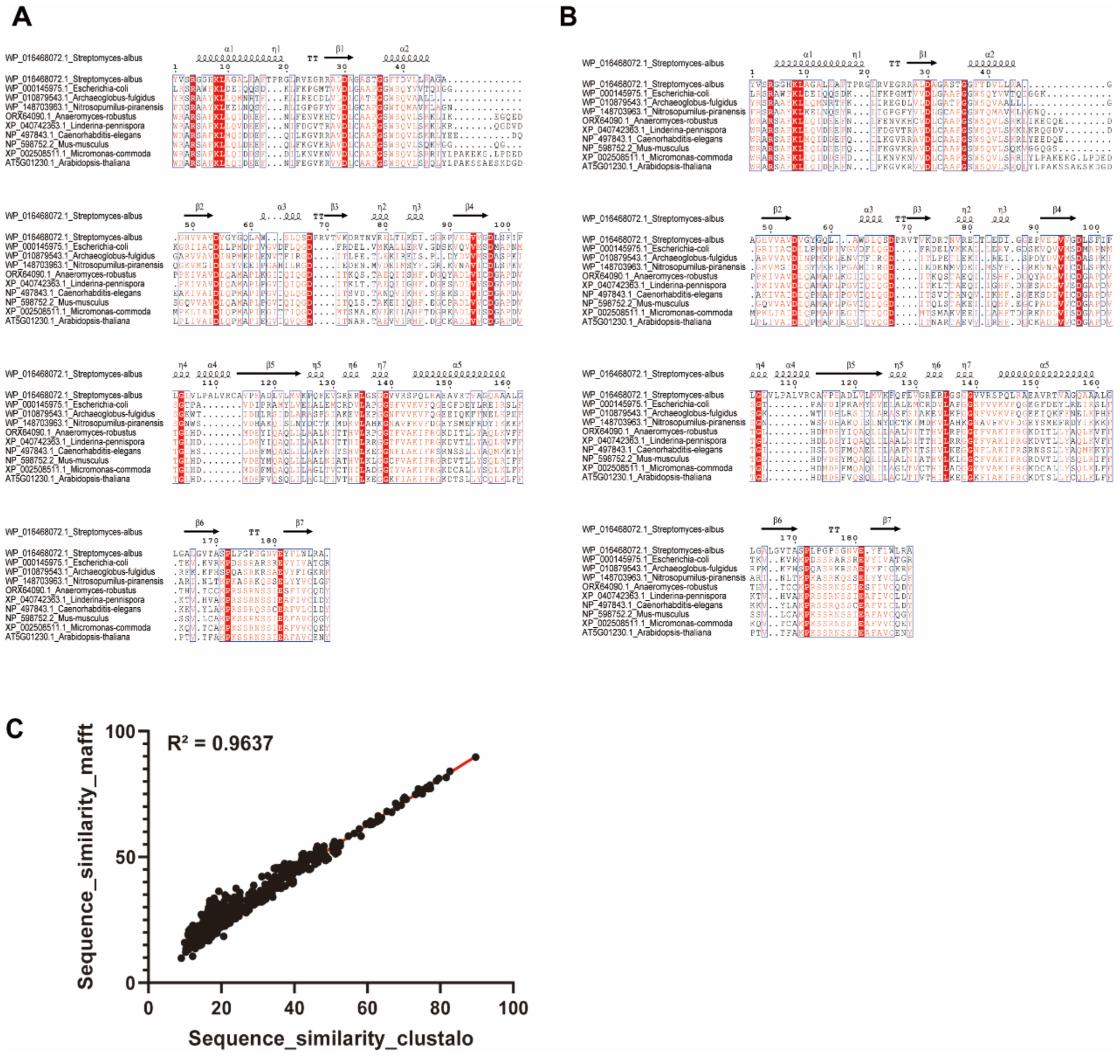

Supplementary Figure S2. Evaluation of multiple sequence alignment quality. A. Scatter plot comparing pairwise sequence identity scores generated using Clustal Omega and MAFFT (L-INS-i), showing high correlation between the two alignment methods. B–C. Representative alignment excerpts of the FtsJ domain visualized using ESPript. B shows the alignment generated by Clustal Omega, and C shows the corresponding alignment from MAFFT. The examples illustrate that alignment gaps are predominantly located in loop or flexible regions, supporting the structural plausibility of the alignments.

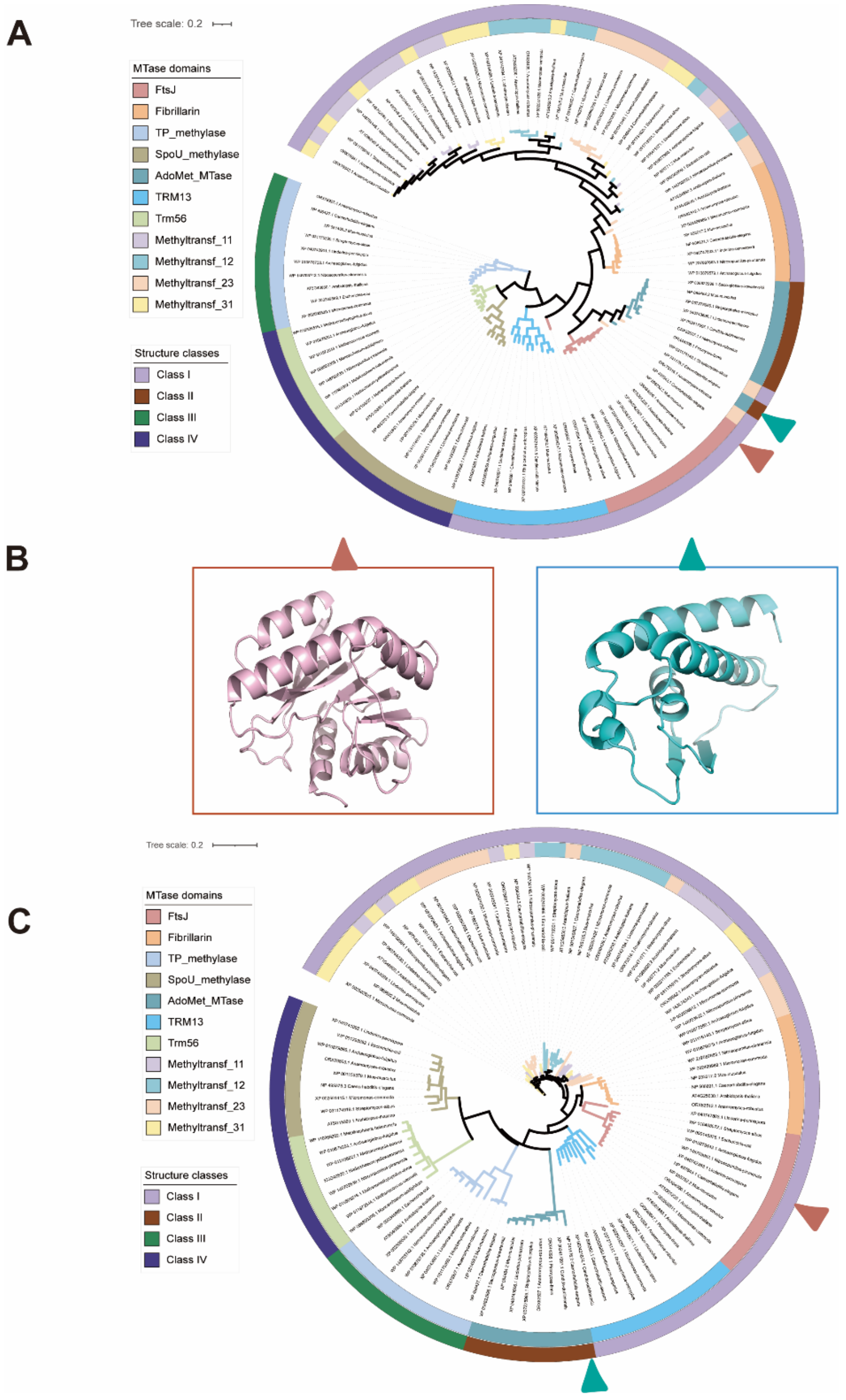

Supplementary Figure S3. Comparison of clustering trees generated by Polyphony and US-align methods. A. Polyphony clustering tree where FtsJ and AdoMet_MTase structures cluster on a single large branch. B. Representative 3D structures of FtsJ (NP_497843.1_Caenorhabditis-elegans_FtsJ) and AdoMet_MTase (NP_741176.2_Caenorhabditis-elegans_AdoMet_MTase) families, highlighting their distinct spatial conformations, particularly in α-helix distribution and β-sheet count. C. US-align clustering tree showing the distinct branches of FtsJ and AdoMet_MTase family proteins. These visual comparisons underscore the differing outcomes of the two clustering methods, with US-align more accurately reflecting the structural divergence relevant to our study on 2′-O-methyltransferases.
